# Influence of Vita Enamic Hybrid Ceramic with Different Thicknesses and Translucencies on the Polymerization of Light-Cured Resin Cement: An *In Vitro* Study

**DOI:** 10.1055/s-0045-1809425

**Published:** 2025-06-17

**Authors:** Esraa Jaber, Emad S. Elsubeihi

**Affiliations:** 1 Department of Clinical Dental Sciences, College of Dentistry, Ajman University, Ajman, United Arab Emirates; 2 Center of Medical and Bio-Allied Health Sciences Research, Ajman University, Ajman, United Arab Emirates

**Keywords:** CAD/CAM, FTIR, Ivocerin, polymer-infiltrated ceramic network (PICN)

## Abstract

**Objectives:**

This study was conducted to evaluate the effect of the CAD/CAM (computer-aided design/computer-aided manufacturing) hybrid ceramic material (Vita Enamic, Vita Zahnfabrik, Bad Säckingen, Germany) in different thicknesses and translucencies on the light irradiance and the obtained radiant exposure, and to measure the Vickers microhardness (HV) and the degree of conversion (DC%) of the light-cured resin cement (Variolink Esthetic LC, Ivoclar Vivadent, Liechtenstein); polymerized by the Vita Enamic samples with different thicknesses and translucencies.

**Materials and Methods:**

The study comprised the polymerization of light-cured resin cement samples through blocks of different thicknesses ranging from 1.0 to 3.0 mm, and two degrees of translucency, namely, translucent and highly translucent Vita Enamic sectional blocks. Light attenuation by the Vita Enamic sections was measured using visible light transmission spectrometry, verifying the accuracy of resin curing (MARC light collector, Bluelight Analytics, Canada). The radiant exposure (RE; in J/cm ^3^ ) or the energy reaching the resin cement samples through Vita Enamic sections during polymerization as well as during polymerization of the resin cement samples directly without the interposition of Vita Enamic sections (control group) was measured. The polymerization efficiency of resin cement was evaluated by using HV and DC% of resin cement using Fourier transform infrared spectroscopy.

**Statistical Analysis:**

The data were analyzed for normality using the Kolmogorov–Smirnov and Shapiro–Wilk tests, which indicated non-normal distribution. Non-parametric Kruskal–Wallis tests were used to determine statistical significance, with Dunn's post-hoc tests for multiple comparisons when significance was detected.Normality tests (Kolmogorov–Smirnov and Shapiro– Wilk) applied to raw data showed that the data were not normally distributed. The nonparametric Kruskal–Wallis test was used to determine statistical significance. If significance was found, multiple comparisons of the groups were tested using Dunn’s post-hoc test.

**Results:**

The results showed a statistically significant decrease in RE (J/cm3), HV, and DC% with increasing thickness of the Vita Enamic sections. On the other hand, despite the decrease in RE (J/cm ^3^ ), HV, and DC% with translucent compared with the same section thickness of the highly translucent Vita Enamic hybrid ceramic, the differences were not statistically significant.

**Conclusion:**

Within the limitations of this study, it can be concluded that increasing the thickness of the Vita Enamic hybrid ceramic sections reduces the light irradiance and the radiant exposure received, as well as the HV and the percentage of conversion of the light-cured resin cement Variolink Esthetic LC of neutral shade. In addition, the different translucency of the Vita Enamic hybrid ceramic, namely, translucent and highly translucent, had a small but nonsignificant effect on the light irradiance and the obtained radiation, as well as on the HV and percentage of conversion of the light-cured resin cement Variolink Esthetic LC of the neutral shade.

## Introduction

Computer-aided design/computer-aided manufacturing (CAD/CAM) restorations have become very popular in esthetic dentistry. As a result, many studies have been conducted on the durability and adhesion of such restorative materials. 1 2 This development has significantly changed the clinical workflow scenario for both dentists and technicians. 3 One of the most important changes is the introduction of monolithic restorations made of high-strength ceramics such as zirconia, or novel ceramic microstructures that have recently been introduced, offering composite and ceramic together with optimized properties. 4 CAD/CAM composites have lower stiffness and hardness compared with ceramics, making them easier to machine using milling machines. This is also beneficial for the opposing dental tissues, which would likely be subject to less wear clinically. In addition, composites are easy to fabricate and repair, and are less brittle than ceramics, resulting in less chipping and cracking during fabrication and aging. 5 According to Mainjot et al, 6 CAD/CAM composite blocks are basically divided into two main categories based on their microstructural geometry: (1) resin with dispersed fillers; (2) polymer-infiltrated ceramic network (PICN). The first category includes composite blocks containing a base monomer type such as Bis-GMA (bisphenol A diglycidylmethacrylate), TEGDMA (triethylene glycol dimethacrylate), and UDMA (urethane dimethacrylate) as an organic matrix with dispersed filler particles such as zirconium dioxide, silicon dioxide, and/or barium glass. 7 PICN materials, on the other hand, consist of a three-dimensional ceramic network infiltrated with a monomer mixture. This blend of composite and ceramic should optimize the performance of these restorative materials by combining the modulus of elasticity of composite, which is similar to dentin, with the long-term esthetic stability of ceramic. 8 PICN is a hybrid dental material combining a sintered ceramic matrix (mostly leucite and some zirconia) with a polymer network. 9 Its flexural strength is generally superior to feldspathic porcelain but lower than lithium disilicate. 9 PICN exhibits damage tolerance to indentation better than some ceramics. Under low loads (up to 200 N), PICN shows high fatigue resistance. 10 However, under higher loads, lithium disilicate and zirconia may perform better. 10 PICN's hardness is lower than ceramics and similar to dental tissues. 11 Its bond strength to resin cements can be influenced by surface treatments, with hydrofluoric acid etching being a common recommendation, although results vary across studies. 12 13 14 PICN's surface roughness after milling is generally between resin composites and feldspathic porcelain. 15 Clinically, PICN crowns have shown promising short-term survival rates. 15 CAD/CAM restorations are usually cemented with resin cement. 16 The popularity of using resin cement for bonding indirect restorations to the tooth structure has increased over the years due to its advantages such as high bond strength, translucency, and shade selection in esthetic areas as well as its low solubility. 17

The composition of the restorative material, translucency, thickness, and shade are the most important factors that can influence the light transmission and thus, the polymerization of resin cement. This finding was confirmed by Pick et al, 18 when they found that the microhardness of dual-cured resin cements was higher than that of light-cured resin cements. They showed that the light transmission through the ceramic material was not sufficient for complete polymerization of the resin and was compensated by the chemical curing in the dual-cured resin cement. 18 In another study by Kilinc et al, 16 it was reported that chemical curing in the dual-cured resin cement could not fully compensate for insufficient polymerization by light. Resin composite polymerization is a form of addition polymerization that produces no by-product. By converting monomers with carbon–carbon double bonds to carbon–carbon single bonds linking one monomer to another, a long chain and interchain crosslink is formed 19 through a process consisting of three steps: induction, propagation, and termination, which cannot be covered in this article. 20 Free radicals are formed by initiators that use either heat (chemical activation of the initiator benzoyl peroxide by tertiary amine) or light energy (either visible or ultraviolet light) to initiate polymerization. Different types of photoinitiators have been used in resin-based composites such as: (1) ultraviolet sensitive photoinitiators; (2) visible light-sensitive photoinitiators which differ in the wavelength at which they absorb light. 21 The photoinitiator Ivocerin—Dibenzoyl-Germanium—has only recently been introduced and is only available in selected products. Recently, this novel dibenzoyl-germanium-based photoinitiator has been added to dental luting cements, which have been reported to exhibit a higher degree of conversion (DC%) and color stability. 22 Inadequate polymerization of light-cured resin materials can lead to many disadvantages, such as increased wear, reduced microhardness, marginal breakdown, and increased postoperative sensitivity. 23 Accordingly, many methods have been mentioned and used in the literature to evaluate the efficacy of polymerization of dental resins, such as microhardness, optical microscopy, scraping back, and Fourier transform infrared spectroscopy (FTIR). 24 25 Many studies have focused on investigating the photopolymerization of these cements under different types of ceramics using different techniques and different light-curing sources. 1 2 16 It is still unclear in the literature that light-cured resin cements can be properly by novel hybrid ceramics and to what extent the thickness or degree of translucency of these restorations may limit their use. Recently, manufacturers have introduced a new type of hybrid ceramic, namely Vita Enamic, a PICN that exhibits high mechanical performance and physical properties. 26 However, there are few studies that have investigated the attenuation of light by this type of hybrid ceramic, the radiant exposure that reaches the resin cement polymerized through this hybrid ceramic, and the efficacy of polymerization and the degree of monomer to polymer conversion of resin cement polymerized through different thicknesses and degrees of translucency of this hybrid ceramic. Therefore, studies are needed to confirm the polymerization efficiency of these Ivocerin-based resin cements through different ceramic materials including Vita Enamic hybrid ceramics.

Therefore, the objectives of this study were to (1) evaluate the effects of the thickness of Vita Enamic hybrid ceramic with different translucencies on the light irradiance and the received radiant exposure on the underside of the light-cured Ivocerin-based resin cement, (2) evaluate the microhardness of the light-cured Ivocerin-based resin cement, used under different thicknesses of Vita Enamic with different translucencies, and (3) to evaluate the DC% of the light-cured Ivocerin-based resin cements used under different thicknesses of Vita Enamic sections with different translucencies. The null hypotheses tested were (1) light irradiance and radiant exposure delivered to light-cured Ivocerin-based resin cement were not affected by increased thickness or increased translucency of the Vita Enamic hybrid ceramic. (2) The microhardness of the light-cured Ivocerin-based resin cement was not affected by the increased thickness or increased translucency of the Vita Enamic hybrid ceramic. (3) The DC% of the light-cured Ivocerin-based resin cement was not affected by increased thickness or increased translucency of the Vita Enamic hybrid ceramic.

## Materials and Methods

**Fig. 1:**
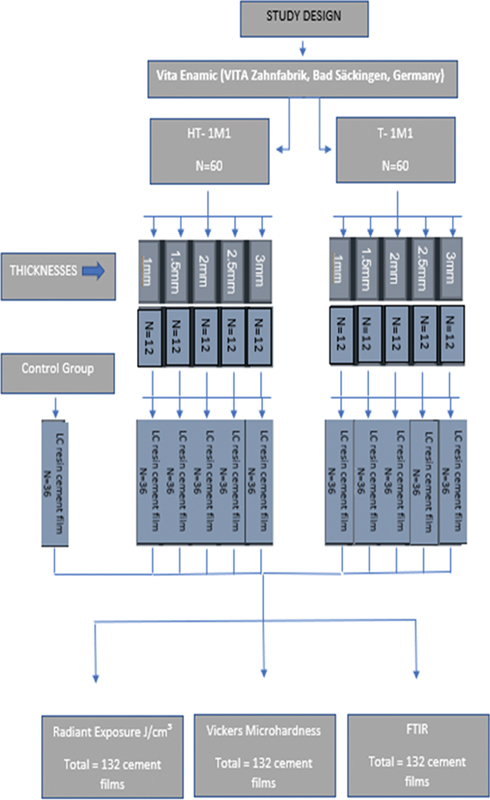
Study design. The figure shows number of hybrid ceramic slabs, number of resin cement films in each group, and tests applied.

The Vita Enamic material used had the shade of 1M1 and two different translucencies namely; highly Translucent (HT) and translucent (LT). For each type of Vita Enamic translucency, sections of five different thicknesses were tested. These were 1.0, 1.5, 2.0, 2.5, and 3.0 mm thick. The minimum sample size was calculated using a power analysis ( *n* = 10/group). In addition, light-curing was performed without interposition of the Vita Enamic sections and used as a control group resulting in 11 groups. The study design is shown in Fig. 1 . The compositions of the investigated CAD/CAM block material and the light-cured resin cement are summarized in Table 1 . The study design involved the polymerization of light-cured resin cement samples through various thicknesses and translucency levels of Vita Enamic sections using an LED light-curing unit (BluePhase N, Ivoclar Vivadent, Liechtenstein). The attenuation of light by the Vita Enamic sections was measured using visible light transmission spectrometry (MARC light collector, Blue-light Analytics, Canada), the radiant exposure (J/cm ^3^ ), or the energy reaching the resin cement samples through the Vita Enamic sections during polymerization, and LED polymerization of resin cement samples directly without the interposition of Vita Enamic sections. The efficiency of the resin cement polymerization, Vickers microhardness (HV), and the DC% of the resin cement were evaluated using FTIR.

**Table 1:** Composition of the materials used in this study

Material	Manufacturer	Classification	Composition	Lot number
Vita Enamic Shade 1M1 (A1) HT, T	VITA Zahnfabrik, Bad Säckingen, Germany	Hybrid ceramic	Aluminum oxide-enriched, fine-structure feldspar matrix (86 wt%, 75 vol%) infused by a polymer material consisting of UDMA and TEGDMA (14 wt%, 25 vol%)	75410
Variolink Esthetic LC (Neutral)	Ivoclar Vivadent, Schaan, Liechtenstein	Light-cured resin cement	Ytterbium trifluoride (10–25%) 1,10-decanediol dimethacrylate (3–10%) Urethane dimethacrylate (3–10%)	666129

### Sample Size Determination

**Fig. 2:**
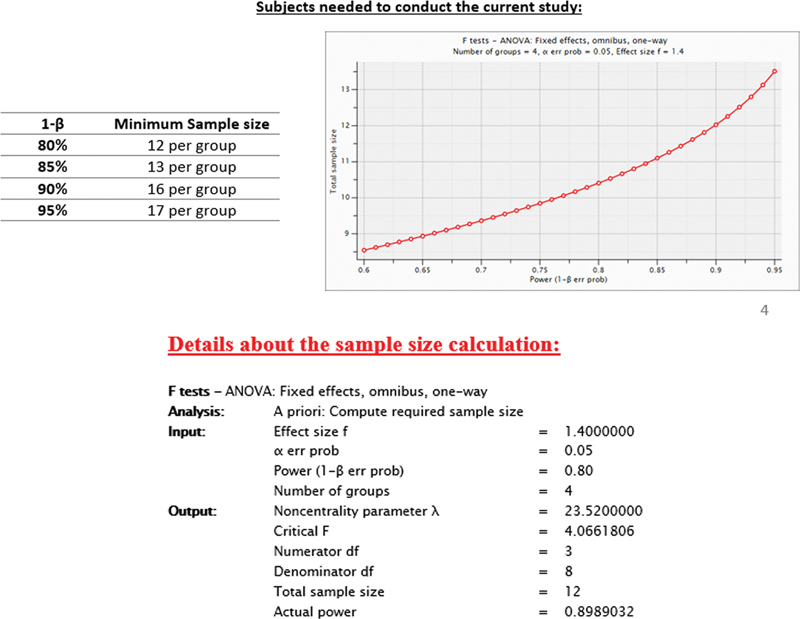
The figure shows the subjects needed to conduct the current study. ( **A** ) Details of sample size calculations.

The sample size of each group was estimated using data available in the literature. 27 The means and standard deviations reported in the literature by Yan et al, were used to estimate the sample size using GPower with the formula:  . This revealed that 12 samples in each group were required to ensure a 5% level of significance ( Fig. 2 and 2A ).

### Production of Hybrid Ceramic Samples

HT and LT Vita Enamic (Vita Zahnfabrik, Bad Säckingen, Germany) CAD/CAM blocks measuring 12 × 14 × 18 mm (shade 1M1) were cut in slabs with a thickness of 1.0, 1.5, 2.0, 2.5, and 3.0 mm using an automated water-cooled low-speed diamond saw (Precision SAW; IsoMet1000, Serial number 713-IPS-04427, BUEHLER, United States).

The resulting Vita Enamic slabs were polished using a grinder and polisher device (MetaServ 250 [twin Grinder-Polisher], serial number 711-MGT-00575, BUEHLER, United States).

**Fig. 3:**
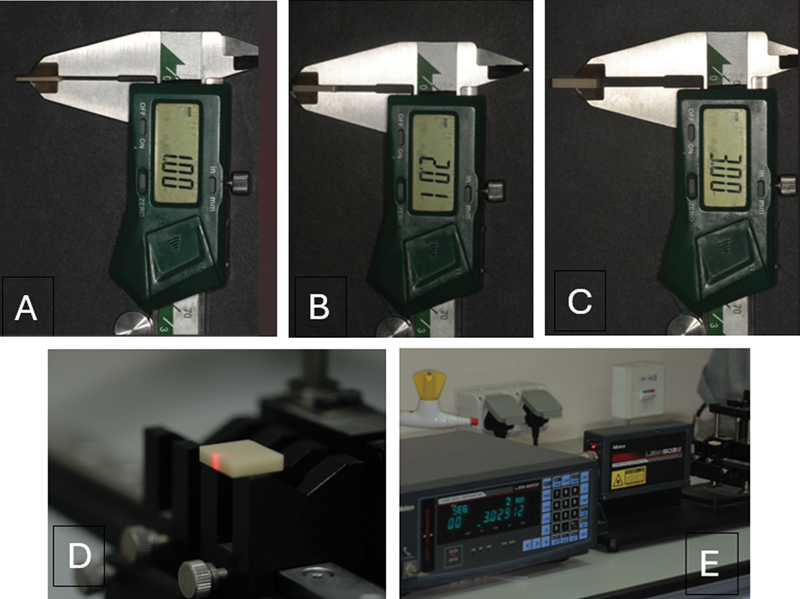
The hybrid ceramic slabs' thicknesses (± 0.01) checked by using Digital Caliper (INSIZE Co., LTD) ( **A** ); desired 1 mm thickness of hybrid ceramic measured with digital caliper ( **B** ); desired 2 mm thickness of hybrid ceramic measured with digital caliper ( **C** ); desired 3 mm thickness of hybrid ceramic measured with digital caliper ( **D** ); 3 mm thickness slab of hybrid ceramic ascertained using laser scan micrometer (LSM-503s, Mitutoyo America Corporation) ( **E** ).

The thickness of the hybrid ceramic slabs was then checked using digital caliper (INSIZE Co., LTD) and the desired thickness (±0.01) was ascertained using laser scanning micrometer (LSM-503s, Mitutoyo America Corporation; Fig. 3 ).

### Preparation of Specimens of Light-Cured Resin Cement

A light-cured resin cement (Variolink Esthetic LC, Ivoclar Vivadent, Liechtenstein) in neutral shade was used in this study. The prepared thickness of the cement was 50 µm in accordance with ISO 4049–2019 28 for the maximum film thickness of luting materials. This thickness was achieved using the following technique. A Mylar strip was placed on the sensor of the MARC resin calibrator, with two perpendicular Mylar strips placed on each end of the base strip. A small drop of cement was placed between the two strips on the base strip. Next, a fourth Mylar strip was placed over the cement drop and the two vertical strips. Then a 50 g weight was placed over the entire unit (the Mylar strips and the cement) for 15 seconds so that the resin cement was compressed to the thickness of the two vertical Mylar strips, which was 50 microns. After the weight was removed, the hybrid ceramic slab was placed on the cement sample unit. A putty silicone light probe jig was then fabricated around the tip of the LED light-curing unit (BluePhase N, Ivoclar Vivadent, Liechtenstein) which was positioned exactly in the center of the hybrid ceramic slab to ensure that the light probe of the light-curing unit was placed in the same position for different hybrid ceramic slabs and resin cement samples. Each group consisted of 12 light-cured resin cement films, resulting in a total of 132 light-cured resin cement films for radiant exposure (J/cm ^3^ ) testing, 132 light-cured resin cement films for microhardness value testing, and 132 light-cured resin cement films for FTIR testing the DC%.

### Light Exposure Time

In this study, an LED light-curing Unit (BluePhase N, Ivoclar Vivadent, Liechtenstein) with a wavelength range of 430 to 490 nm and a light intensity of 1,200 mW/cm ^2^ , equipped with a 10 mm curved light probe, was used. In the manufacturer's instructions, the curing time of Variolink Esthetic LC (Ivoclar Vivadent, Liechtenstein) is specified as 10 seconds per millimeter of ceramic. The manufacturer of Vita Enamic recommends the use of a high-power LED, therefore 10–15–20–25–30 seconds of high-power LED with 1,200 mW/cm ^2^ intensity was chosen for this study depending on the thickness of ceramic being used.

### Measurement of Light Attenuation by the Hybrid Ceramic Slabs

**Fig. 4:**
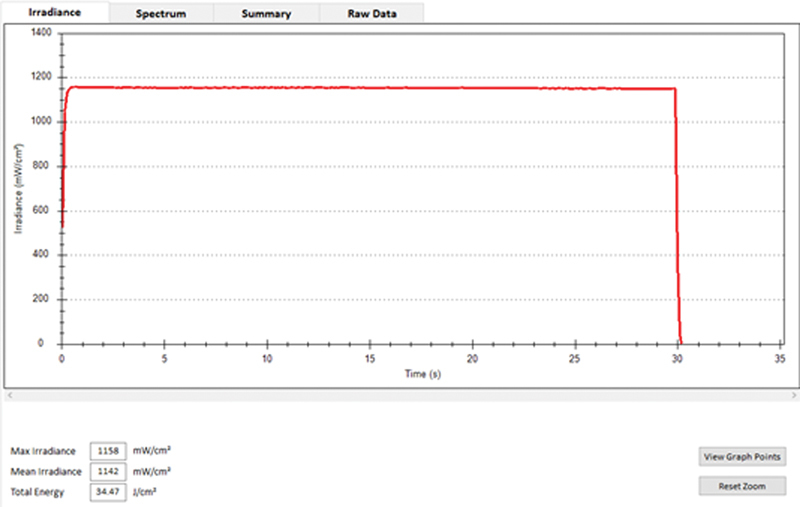
Graph demonstrates the irradiance (mW/cm ^2^ ) delivered to the sensor on the MARC during the time period of light exposure.

**Fig. 5:**
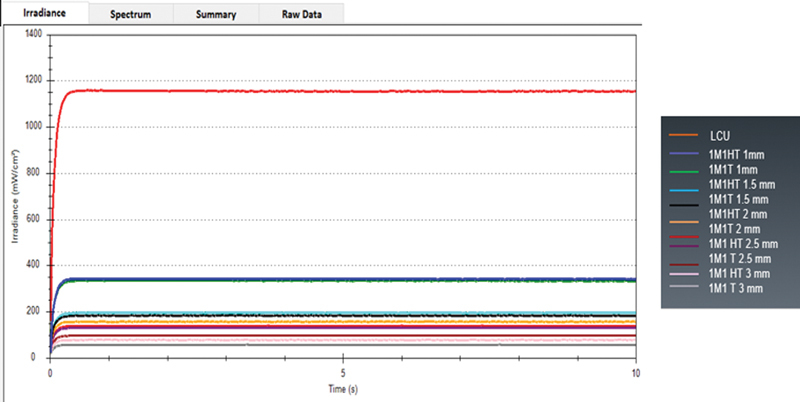
Graph demonstrates the irradiance (mW/cm ^2^ ) delivered to the sensor through all samples of different thicknesses and translucencies on the MARC during the time period of light exposure.

The experiment was performed using light transmission spectrometry in the visible range (MARC light collector, Bluelight Analytics, Canada). The sensor in the MARC resin calibrator is connected to a spectrometer. The spectrometer was used to measure both the irradiance and spectral irradiance of the light source alone and through different Vita Enamic hybrid ceramic slabs ( Figs. 4 and 5 ). The silicone light probe guide was used on all samples to ensure the central position of the light probe over each sample. The radiant exposure of resin cement samples polymerized under different hybrid ceramic slabs and through the cement sample directly without interposing a hybrid ceramic slab between the light source and the resin cement sample (control group) was recorded by the spectrometer. The radiant exposure (J/cm ^3^ ) is the total area under the spectral curve in the corresponding graph of each test. The information is calculated and automatically stored in the software.

After curing with LED light, the samples were kept between the Mylar strips, the bottom surface of each film was marked, and the entire unit of Mylar strips and resin cement samples was stored for 24 hours at 37°C in an incubator in a dark environment.

### Measuring the Polymerization Efficiency of Light-Cured Resin Cements by Microhardness

The standard test method for Knoop and Vickers hardness (HV) of materials (ASTM (E384–11–1) recommends that the load must be such that the penetration depth of the pyramid into the coating is no more than 1/10 of the coating thickness. Therefore, the appropriate indentation force for the 50-micron thick sample during the microhardness test was calculated using the formula *d* = 7 *t* , where *d* = mean diagonal length and *t* = penetration depth. Each sample was tested for HV using a microhardness tester (Future-Tech Corp., model FM-800, serial number FMX8346, Japan) with a Vickers indenter with a force of 5 ngF and 10 seconds dwell time ( Table 2 ).

**Table 2:** Comparison of the indentations produced with three different test loads (5, 10, and 25 gF) on specimens that were cured through the thickest low translucent hybrid ceramic slab (translucent: 3 mm thick)

1M1 T 3 mm	5 g	10 g	25 g
Resin cement specimen of 50-micron thickness Penetration depth	5.4 µm	9.5 µm	17.5 µm

### Measurement of the Degree of Conversion

The samples were analyzed using FTIR (IRAffinity-1S, Shimadzu Corp., Japan) with an attenuated total reflectance (ATR) measurement accessory to determine the DC%. The FTIR spectra were collected for a sample of unreacted monomer as well as for the polymerized samples through Vita Enamic slabs. The measurements of the DC% were performed at 22°C (room temperature) and at a relative humidity of 50% to avoid premature setting of the cement due to ambient light. The infrared spectra were acquired from the bottom of the sample in the wavelength range of 4,000–500 cm ^−1^ , a wavenumber resolution of 4 cm ^−1^ , and a rate of 45 scans/spectrum as measurement parameters in absorbance mode. A single measurement was performed for each of the 132 samples. As a negative control, a drop of uncured cement was applied to the ATR crystal and infrared spectra were recorded from the underside of the sample without light irradiation to determine the chemical curing of the composite cement. The DC% was calculated from the aliphatic C = C peak at 1,638 cm ^−1^ and normalized against the aromatic C = C peak at 1,608 cm ^−1^ , using the following equation:

DC% = [1 − ( *A*_1,638_ / *A*_1,608_ ) _cured_ /( *A*_1,638_ / *A*_1,608_ ) _uncured_ ] × 100

where:

- A1638A_ {1638}A1638 = absorbance of the *aliphatic* C = C bond at 1,638 cm ^−1^ .
- A1608A_ {1608}A1608 = absorbance of the *aromatic* C = C bond at 1,608 cm ^−1^ .
- The subscript “cured” refers to the polymerized specimen, and “uncured” refers to the unpolymerized material.

The conversion level of each sample was calculated as a percentage.

## Results

### Measured Variables

The variables measured in this study included the radiant exposure (J/cm ^3^ ), microhardness, and the DC% of light-cured cement (Variolink Esthetic LC) cured under different thicknesses (1.0, 1.5, 2.0, 2.5, and 3.0 mm) of thick slabs with the translucency levels (HT, T) and shade 1M1. Eleven groups including the control group were evaluated for each variable.

### Statistical Analysis

Normality tests (Kolmogorov–Smirnov and Shapiro–Wilk) were applied to the raw data. The analysis of the normality tests showed that the data were not normally distributed. Nonparametric tests were used to test for statistical significance. Radiant exposure, microhardness value, and DC% were analyzed using the nonparametric test (Kruskal–Wallis). If a significant difference was found between the groups, multiple comparisons of the groups were revealed using the Dunn's test. A *p* -value of ≤0.05 was considered statistically significant. Statistical analysis was performed using SPSS (version 21, 64-bit edition, IBM, United States).

### Comparison of Radiant Exposure (J/cm ^3^ ), Microhardness, and Degree of Conversion between All Groups

The mean values and standard deviations of radiant exposure (microhardness and DC%—after 24 hours of incubation at 37°C) of the control group and for the samples polymerized under (1.0, 1.5, 2.0, 2.5, and 3.0 mm) HT and LT are shown in ( Tables 3 4 5 ).

**Table 3:** Descriptive statistics of radiant exposure (in J/cm ^3^ ) of all groups tested

Sample No.	High translucency					Low translucency					Control
	Thickness of hybrid ceramic					Thickness of hybrid ceramic					
	1.0 mm	1.5 mm	2.0 mm	2.5 mm	3.0 mm	1.0 mm	1.5 mm	2.0 mm	2.5 mm	3.0 mm	
**1**	**9**	**5.77**	**3.72**	**2.61**	**1.81**	**7.25**	**3.47**	**2.29**	**2.27**	**1.6**	**15.98**
2	7.6	4.46	3.67	2.56	2.22	6.82	3.53	2.58	2.13	1.52	22.14
3	9.42	5.25	3.65	2.22	2.24	6.18	2.36	2.38	2.24	1.28	15.35
4	8.75	5.59	3.61	2.26	1.39	6.29	2.17	2.53	2.02	1.43	21.94
5	8.92	5.3	3.95	2.45	2.16	6.98	2.31	2.69	2.05	1.43	23.15
6	8.74	5.07	3.81	2.48	1.98	6.62	2.31	2.57	2.09	1.28	17.54
7	8.99	4.97	3.31	2.47	1.31	6.98	2.37	2.4	1.86	1.22	17.45
8	8.33	5.1	3.53	2.38	1.35	7.17	2.48	2.64	2.03	1.3	15.26
9	7.96	4.99	3.59	2.33	1.81	7.24	2.43	2.5	1.89	1.44	16.34
10	8.54	4.17	3.5	2.51	1.95	6.12	2.25	2.62	1.97	1.26	20.56
11	9.77	5.66	3.59	2.47	1.67	7.5	2.87	2.5	1.78	1.31	18.45
12	8.54	6.72	3.24	2.59	1.89	5.9	2.55	2.3	2.06	1.32	16.91
**Mean**	**8.713**	**5.254**	**3.597**	**2.444**	**1.815**	**6.754**	**2.591**	**2.5**	**2.032**	**1.365**	**18.422**
**SD**	**0.590**	**0.654**	**0.194**	**0.124**	**0.328**	**0.523**	**0.459**	**0.131**	**0.1449**	**0.116**	**2.810**

Abbreviation: SD, standard deviation.

**Table 4:** Descriptive statistics of microhardness value (HV) of all groups tested

Sample No.	High translucency (HT)					Translucent (LT)					Control
	Thickness of hybrid ceramic					Thickness of hybrid ceramic					
	1.0 mm	1.5 mm	2.0 mm	2.5 mm	3.0 mm	1.0 mm	1.5 mm	2.0 mm	2.5 mm	3.0 mm	
**1**	8.6	6.3	4.8	4	4.7	7.7	7.1	4.7	2.3	5.8	8.5
**2**	4.4	4.6	5.4	6.2	5.8	5.6	4.6	5	3.9	3.1	9.8
**3**	8.1	5.2	4.3	4.4	4.4	6.4	5	5	5.5	3.4	8.5
**4**	9	4.4	5.5	4.1	4.4	6.4	6.8	5.1	6.8	3	9.3
**5**	8.9	6	5.9	4.9	5.7	7.2	3.6	4.5	3.2	2.8	7.9
**6**	8.7	4.3	4.6	4.8	4.2	5.2	4.3	4.9	6.4	3.4	8
**7**	6.9	5.9	5.7	4.7	4.6	8.3	3.9	5.5	4.4	1.5	8.1
**8**	8.4	6.5	5.5	5.8	3.4	10	5.7	5.6	3.2	3	10
**9**	6.8	6	5.3	5.1	4.1	7.2	3.6	5.4	4.3	3.2	8.7
**10**	5.3	5.5	5.5	5.5	2.7	7.8	3.6	4.5	5	6.2	9.3
**11**	6	6.4	5.7	6.3	3.7	6	7.8	4.4	6.4	5.6	7.9
**12**	5.9	6.5	5.5	5	4	6.2	5.1	5.1	7.2	2.5	7.3
**Mean**	**7.25**	**5.633**	**5.308**	**5.066**	**4.308**	**7**	**5.091**	**4.975**	**4.883**	**3.625**	**8.608**
**SD**	**1.577**	**0.822**	**0.485**	**0.755**	**0.871**	**1.330**	**1.465**	**0.398**	**1.593**	**1.447**	**0.836**

Abbreviation: SD, standard deviation.

**Table 5:** Descriptive statistics of percent degree of conversion (DC%) of all groups tested

Sample No.	High translucency					Low translucency					Control
	Thickness of hybrid ceramic					Thickness of hybrid ceramic					
	1.0 mm	1.5 mm	2.0 mm	2.5 mm	3.0 mm	1.0 mm	1.5 mm	2 mm	2.5 mm	3.0 mm	
1	83	74.3	66.8	41.9	35.3	89.3	63.6	54.5	32.3	15.5	95.3
2	79.7	67.2	53.7	28.4	25.6	79.7	71.8	55.4	35.2	20	96.7
3	91.7	69.6	85.9	35.4	33.1	78.4	73	67.8	22.9	26.6	94.4
4	81	67.9	69.4	36.8	28	88.4	67.9	63.4	35.2	30.5	95.6
5	83.2	87.5	71.2	31.4	35.6	84.7	77.2	61	20.1	26.1	95.6
6	93.5	74.8	62.7	32	29.8	89.3	74.3	56.6	26	24.4	93.5
7	88	84.4	54.3	39.2	23	84.2	71.8	59.5	26	20.2	97.8
8	83.5	83.7	62.2	28.5	21.3	80.4	75.1	69.9	20	20.7	96.7
9	87.6	80.6	65.4	31.3	37.3	87.5	77.2	56.4	38.3	23.1	93.7
10	86.1	71.2	52.8	31.6	30.2	88.3	71.8	62.9	25.9	21.7	94.9
11	88.6	62.6	75.2	38.1	23.2	88.3	73.1	58.8	29.2	19.4	95.3
12	78.2	66.9	71.6	31.1	22.9	74.8	60.1	53.3	36.2	19.1	93.7
**Mean**	**85.34**	**74.22**	**65.93**	**33.80**	**28.77**	**84.44**	**71.40**	**59.95**	**28.94**	**22.27**	**95.266**
**SD**	**4.724**	**8.085**	**9.720**	**4.363**	**5.633**	**4.958**	**5.188**	**5.242**	**6.404**	**4.075**	**1.337**

Abbreviation: SD, standard deviation.

The Kruskal–Wallis statistics showed a highly significant difference between the tested groups for radiant exposure and microhardness as well as for the DC% ( *p* = 0.0001). Multiple comparisons of the groups were revealed by Dunn's post-hoc tests.

A significant reduction in radiant exposure was found between 1.0 mm and all other thicknesses (1.5, 2.0, 2.5, and 3.0 mm) of hybrid ceramic.

Within the two (HT, T) groups, no significant difference was found between the 1.0 mm thick hybrid ceramic and the control group in terms of radiant exposure, microhardness, and DC ( *p* = 0.118, *p* = 0.161, *p* = 0.187), respectively. Radiant exposure (J/cm ^3^ ), HV, and DC decreased with increasing thickness. Despite the fact that radiant exposure, HV, and DC decreased with increasing thickness from 2.0 to 2.5 mm and with increasing thickness from 2.5 to 3.0 mm, the differences were not statistically significant ( Tables 6 7 8 9 10 11 ).

**Table 6:** Dunn's statistic results of comparison of radiant exposure (J/cm ^3^ ) based on thickness among high translucency hybrid ceramic group based on Dunn's statistics

	Sample	Test statistic	Std. error	Std. test statistic	Sig.
**Thickness 3.0 mm HT**	**2.5 mm HT**	19.625	15.615	1.257	0.209
	**2.0 mm HT**	49.417	15.615	3.165	0.002
	**1.5 mm HT**	45.208	15.615	2.895	0.004
	**1.0 mm HT**	94.208	15.615	6.033	0.000
	**Control**	118.625	15.615	7.597	0.000
**Thickness 2.5 mm HT**	**3.0 mm HT**	19.625	15.615	1.257	0.209
	**2.0 mm HT**	29.792	15.615	1.908	0.056
	**1.5 mm HT**	25.583	15.615	1.638	0.001
	**1.0 mm HT**	74.583	15.615	4.776	0.000
	**Control**	99.000	15.615	6.340	0.000
**Thickness 2.0 mm HT**	**3.0 mm HT**	49.417	15.615	3.165	0.002
	**2.5 mm HT**	29.792	15.615	1.908	0.056
	**1.5 mm HT**	−4.208	15.615	−0.270	0.009
	**1.0 mm HT**	44.792	15.615	2.869	0.004
	**Control**	69.208	15.615	4.432	0.000
**Thickness 1.5 mm HT**	**3.0 mm HT**	45.208	15.615	2.895	0.004
	**2.5 mm HT**	25.583	15.615	1.638	0.001
	**2.0 mm HT**	−4.208	15.615	−0.270	0.009
	**1.0 mm HT**	49.000	15.615	3.138	0.002
	**Control**	73.417	15.615	4.702	0.000
**Thickness 1.0 mm HT**	**3.0 mm HT**	94.208	15.615	6.033	0.000
	**2.5 mm HT**	74.583	15.615	4.776	0.000
	**2.0 mm HT**	44.792	15.615	2.869	0.004
	**1.5 mm HT**	49.000	15.615	3.138	0.002
	**Control**	24.417	15.615	1.564	0.118

**Table 7:** Dunn's statistic results of comparison of radiant exposure (J/cm ^3^ ) based on thickness among low translucency hybrid ceramic group based on Dunn's statistics

	Sample	Test statistic	Std. error	Std. test statistic	Sig.
**Thickness 3.0 mm LT**	**2.5 mm LT**	31.875	15.615	2.041	0.241
	**2.0 mm LT**	57.125	15.615	3.658	0.000
	**1.5 mm LT**	70.000	15.615	4.483	0.000
	**1.0 mm LT**	93.583	15.615	5.993	0.000
	**Control**	105.583	15.615	6.762	0.000
**Thickness 2.5 mm LT**	**3.0 mm LT**	31.875	15.615	2.041	0.241
	**2.0 mm LT**	25.250	15.615	1.617	0.106
	**1.5 mm LT**	38.125	15.615	2.442	0.015
	**1.0 mm LT**	61.708	15.615	3.952	0.000
	**Control**	73.708	15.615	4.720	0.000
**Thickness 2.0 mm LT**	**3.0 mm LT**	57.125	15.615	3.658	0.000
	**2.5 mm LT**	25.250	15.615	1.617	0.106
	**1.5 mm LT**	12.875	15.615	0.825	0.041
	**1.0 mm LT**	36.458	15.615	2.335	0.020
	**Control**	48.458	15.615	3.103	0.002
**Thickness 1.5 mm LT**	**3.0 mm LT**	70.000	15.615	4.483	0.000
	**2.5 mm LT**	38.125	15.615	2.442	0.015
	**2.0 mm LT**	12.875	15.615	0.825	0.041
	**1.0 mm LT**	53.893	15.615	2.098	0.000
	**Control**	75.578	15.615	4.980	0.000
**Thickness 1.0 mm LT**	**3.0 mm LT**	93.583	15.615	5.993	0.000
	**2.5 mm LT**	61.708	15.615	3.952	0.000
	**2.0 mm LT**	12.875	15.615	0.825	0.041
	**1.5 mm LT**	53.893	15.615	2.098	0.000
	**Control**	24.417	15.615	1.564	0.118

**Table 8:** Dunn's statistic results of comparison of microhardness values (HV) based on thickness among high translucency (HT) Vita Enamic hybrid ceramic groups

	Sample	Test statistic	Std. error	Std. test statistic	Sig.
**Thickness 3.0 mm HT**	**2.5 mm HT**	26.292	15.609	1.684	0.092
	**2.0 mm HT**	25.958	15.609	1.663	0.096
	**1.5 mm HT**	27.583	15.609	1.767	0.008
	**1.0 mm HT**	73.667	15.609	4.719	0.000
	**Control**	95.542	15.609	6.121	0.000
**Thickness 2.5 mm HT**	**3.0 mm HT**	26.292	15.609	1.684	0.092
	**2.0 mm HT**	−0.333	15.609	−0.021	0.983
	**1.5 mm HT**	1.292	15.609	0.083	0.034
	**1.0 mm HT**	47.375	15.609	3.035	0.002
	**Control**	69.250	15.609	4.436	0.000
**Thickness 2.0 mm HT**	**3.0 mm HT**	25.958	15.609	1.663	0.096
	**2.5 mm HT**	−0.333	15.609	−0.021	0.983
	**1.5 mm HT**	1.625	15.609	0.104	0.017
	**1.0 mm HT**	47.708	15.609	3.056	0.002
	**Control**	69.583	15.609	4.458	0.000
**Thickness 1.5 mm HT**	**3.0 mm HT**	27.583	15.609	1.767	0.008
	**2.5 mm HT**	1.292	15.609	0.083	0.034
	**2.0 mm HT**	1.625	15.609	0.104	0.017
	**1.0 mm HT**	46.083	15.609	2.952	0.003
	**Control**	67.958	15.609	4.354	0.000
**Thickness 1.0 mm HT**	**3.0 mm HT**	73.667	15.609	4.719	0.000
	**2.5 mm HT**	47.375	15.609	3.035	0.002
	**2.0 mm HT**	47.708	15.609	3.056	0.002
	**1.5 mm HT**	46.083	15.609	2.952	0.003
	**Control**	21.875	15.609	1.401	0.161

**Table 9:** Dunn's statistic results of comparison of microhardness values (HV) based on thickness among translucent (LT) Vita Enamic hybrid ceramic groups

	Sample	Test statistic	Std. error	Std. test statistic	Sig.
**Thickness 3.0 mm LT**	**2.5 mm LT**	20.458	15.609	1.311	0.190
	**2.0 mm LT**	28.208	15.609	1.807	0.071
	**1.5 mm LT**	38.792	15.609	2.485	0.013
	**1.0 mm LT**	66.500	15.609	4.260	0.000
	**Control**	87.042	15.609	5.576	0.000
**Thickness 2.5 mm LT**	**3.0 mm LT**	20.458	15.609	1.311	0.190
	**2.0 mm LT**	7.750	15.609	0.496	0.020
	**1.5 mm LT**	18.333	15.609	1.175	0.024
	**1.0 mm LT**	46.042	15.609	2.950	0.003
	**Control**	66.583	15.609	4.266	0.000
**Thickness 2.0 mm LT**	**3.0 mm LT**	28.208	15.609	1.807	0.071
	**2.5 mm LT**	7.750	15.609	0.496	0.020
	**1.5 mm LT**	10.583	15.609	0.678	0.050
	**1.0 mm LT**	38.292	15.609	2.453	0.014
	**Control**	58.833	15.609	3.769	0.000
**Thickness 1.5 mm LT**	**3.0 mm LT**	38.792	15.609	2.485	0.013
	**2.5 mm LT**	18.333	15.609	1.175	0.024
	**2.0 mm LT**	10.583	15.609	0.678	0.050
	**1.0 mm LT**	27.708	15.609	1.775	0.046
	**Control**	48.250	15.609	3.091	0.002
**Thickness 1.0 mm LT**	**3.0 mm LT**	66.500	15.609	4.260	0.000
	**2.5 mm LT**	46.042	15.609	2.950	0.003
	**2.0 mm LT**	38.292	15.609	2.453	0.014
	**1.5 mm LT**	27.708	15.609	1.775	0.046
	**Control**	20.542	15.609	1.316	0.188

**Table 10:** Dunn's statistic results of comparison of percent degree of conversion (DC%) based on thickness among high translucency Vita Enamic hybrid ceramic groups

	Sample	Test statistic	Std. error	Std. test statistic	Sig.
**Thickness 3.0 mm HT**	**2.5 mm HT**	13.792	15.615	0.883	0.377
	**2.0 mm HT**	48.458	15.615	3.103	0.002
	**1.5 mm HT**	68.958	15.615	4.416	0.000
	**1.0 mm HT**	94.417	15.615	6.047	0.000
	**Control**	115.000	15.615	7.365	0.000
**Thickness 2.5 mm HT**	**3.0 mm HT**	13.792	15.615	0.883	0.377
	**2.0 mm HT**	34.667	15.615	2.220	0.066
	**1.5 mm HT**	55.167	15.615	3.533	0.000
	**1.0 mm HT**	80.625	15.615	5.163	0.000
	**Control**	101.208	15.615	6.482	0.000
**Thickness 2.0 mm HT**	**3.0 mm HT**	48.458	15.615	3.103	0.002
	**2.5 mm HT**	34.667	15.615	2.220	0.066
	**1.5 mm HT**	20.500	15.615	1.313	0.009
	**1.0 mm HT**	45.958	15.615	2.943	0.003
	**Control**	66.542	15.615	4.261	0.000
**Thickness 1.5 mm HT**	**3.0 mm HT**	68.958	15.615	4.416	0.000
	**2.5 mm HT**	55.167	15.615	3.533	0.000
	**2.0 mm HT**	20.500	15.615	1.313	0.009
	**1.0 mm HT**	20.517	15.615	1.987	0.018
	**Control**	41.917	15.615	2.684	0.007
**Thickness 1.0 mm HT**	**3.0 mm HT**	94.417	15.615	6.047	0.000
	**2.5 mm HT**	80.625	15.615	5.163	0.000
	**2.0 mm HT**	45.958	15.615	2.943	0.003
	**1.5 mm HT**	20.517	15.615	1.987	0.018
	**Control**	20.583	15.615	1.318	0.187

**Table 11:** Dunn's statistic results of comparison of percent degree of conversion (DC%) based on thickness among translucent (LT) Vita Enamic hybrid ceramic groups

	Sample	Test statistic	Std. error	Std. test statistic	Sig.
Thickness 3.0 mm LT	2.5 mm LT	10.708	15.615	0.686	0.493
	2.0 mm LT	44.583	15.615	2.855	0.004
	1.5 mm LT	59.250	15.615	3.794	0.003
	1.0 mm LT	81.125	15.615	5.195	0.000
	Control	101.167	15.615	6.479	0.000
Thickness 2.5 mm LT	3.0 mm LT	10.708	15.615	0.686	0.493
	2.0 mm LT	33.875	15.615	2.169	0.030
	1.5 mm LT	48.542	15.615	3.109	0.002
	1.0 mm LT	70.417	15.615	4.510	0.000
	Control	90.458	15.615	5.793	0.000
Thickness 2.0 mm LT	3.0 mm LT	44.583	15.615	2.855	0.004
	2.5 mm LT	33.875	15.615	2.169	0.030
	1.5 mm LT	14.667	15.615	0.939	0.035
	1.0 mm LT	36.542	15.615	2.340	0.019
	Control	56.583	15.615	3.624	0.000
Thickness 1.5 mm LT	3.0 mm LT	59.250	15.615	3.794	0.003
	2.5 mm LT	48.542	15.615	3.109	0.002
	2.0 mm LT	14.667	15.615	0.939	0.035
	1.0 mm LT	20.517	15.615	1.987	0.018
	Control	41.917	15.615	2.684	0.007
Thickness 1.0 mm LT	3.0 mm LT	81.125	15.615	5.195	0.000
	2.5 mm LT	70.417	15.615	4.510	0.000
	2.0 mm LT	36.542	15.615	2.340	0.019
	1.5 mm LT	20.517	15.615	1.987	0.018
	Control	20.042	15.615	1.284	0.199

**Fig. 6:**
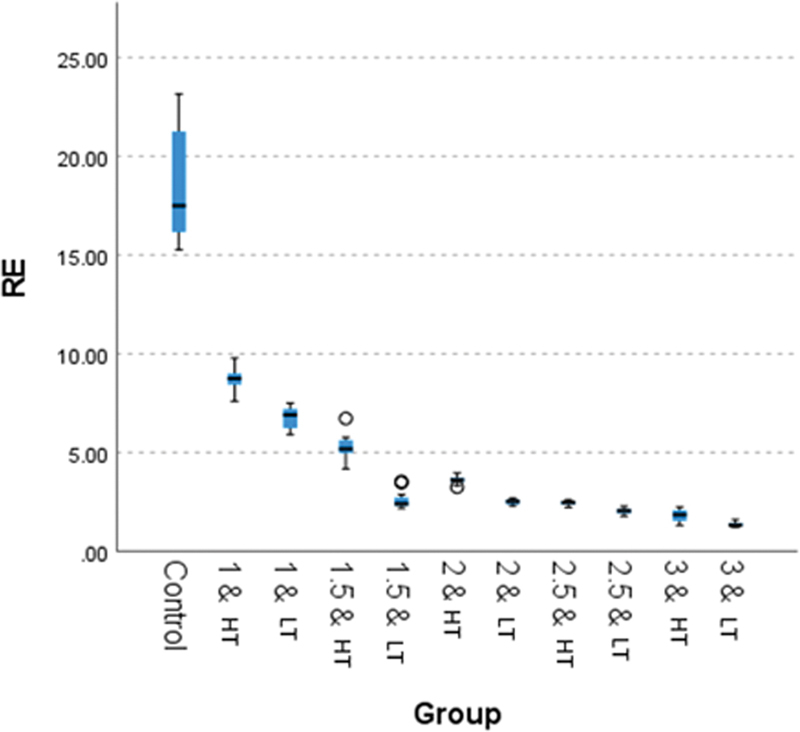
Boxplots of radiant exposure data (in J/cm ^3^ ) of 50-micron thickness light-cured resin cement polymerized without interposition of hybrid ceramic slab (control), and light-cured resin cement polymerized under 1.0, 1.5, 2.0, 2.5, and 3.0 mm of hybrid ceramic slab of translucent (LT) and highly translucent (HT) types.

Comparison of the corresponding thickness of LT and HT Vita Enamic demonstrated that there was decreased radiant exposure in LT as compared with HT Vita Enamic of radiant exposure passing through the same corresponding section thickness. Furthermore, as the thickness of LT was increased to 2.0, 2.5, and 3.0 mm, the radiant exposure was reduced in comparison to the corresponding HT thicknesses, but the differences did not reach statistical significance ( Table 12 and Fig. 6 ).

**Table 12:** Dunn's statistic results of comparison of radiant exposure (J/cm ^3^ ) among high translucent (HT) and translucent (LT) Vita Enamic hybrid ceramic groups

	Sample	Test statistic	Std. error	Std. test statistic	Sig.
Thickness 3.0 mm LT	3.0 mm HT	13.042	15.615	0.835	0.404
	2.5 mm HT	22.436	15.615	3.593	0.304
	2.0 mm HT	50.581	15.615	6.483	0.008
	1.5 mm HT	47.248	15.615	3.815	0.003
	1.0 mm HT	106.625	15.615	6.828	0.000
Thickness 2.5 mm LT	3.0 mm HT	24.951	15.615	2.098	0.324
	2.5 mm HT	25.292	15.615	1.620	0.105
	2.0 mm HT	31.908	15.615	2.091	0.068
	1.5 mm HT	27.502	15.615	2.937	0.003
	1.0 mm HT	75.745	15.615	4.987	0.000
Thickness 2.0 mm LT	3.0 mm HT	54.581	15.615	2.098	0.006
	2.5 mm HT	23.512	15.615	1.309	0.113
	2.0 mm HT	20.750	15.615	1.329	0.184
	1.5 mm HT	13.093	15.615	1.072	0.034
	1.0 mm HT	445.809	15.615	3.009	0.000
Thickness 1.5 mm LT	3.0 mm HT	65.203	15.615	3.980	0.000
	2.5 mm HT	35.798	15.615	2.304	0.016
	2.0 mm HT	36.145	15.615	3.109	0.004
	1.5 mm HT	37.833	15.615	2.423	0.015
	1.0 mm HT	50.809	15.615	3.245	0.000
Thickness 1.0 mm LT	3.0 mm HT	101.034	15.615	4.209	0.000
	2.5 mm HT	59.642	15.615	3.509	0.000
	2.0 mm HT	34.723	15.615	2.795	0.000
	1.5 mm HT	53.532	15.615	1.903	0.000
	1.0 mm HT	12.000	15.615	0.795	0.427

**Fig. 7:**
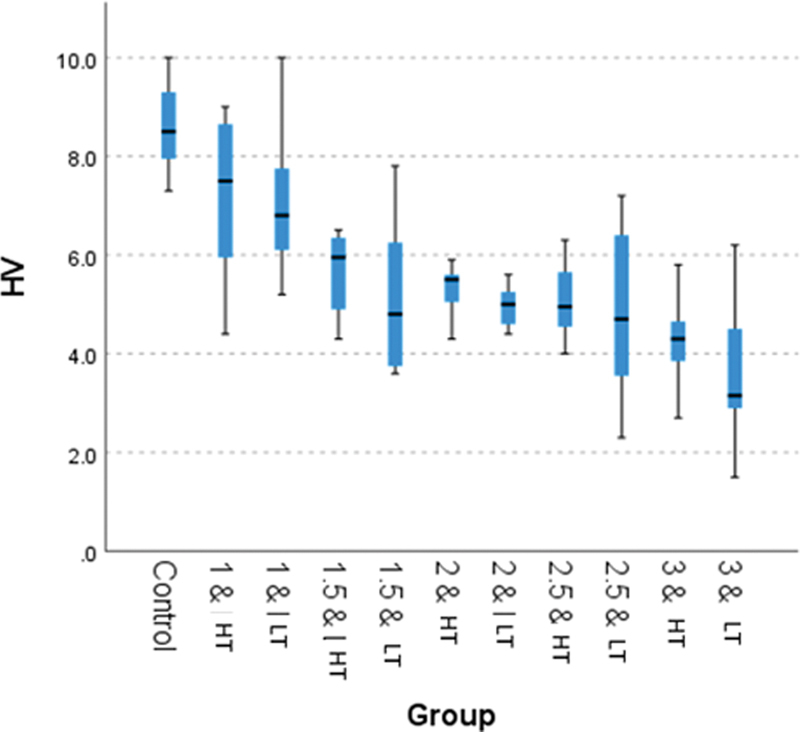
Boxplots of microhardness value (HV) of 50-micron thickness light-cured resin cement polymerized without interposition of hybrid ceramic slab (control), and light-cured resin cement polymerized under 1.0, 1.5, 2.0, 2.5, and 3.0 mm of hybrid ceramic slab of translucent and highly translucent types.

Comparison of the corresponding thickness of LT and HT Vita Enamic demonstrated that there was decreased HV in LT as compared with HT Vita Enamic. Furthermore, analysis revealed no statistical significance between 1.0 mm HT and 1.0 mm LT hybrid ceramic ( *p* = 0.427), as the thickness of LT was increased to1.5, 2.0, 2.5, and 3.0 mm, the HV was reduced in comparison to the corresponding HT thicknesses, but the differences did not reach statistical significance ( Table 13 and Fig. 7 ).

**Table 13:** Dunn's statistic results of comparison of microhardness values (HV) among high (HT) and translucent (LT) hybrid ceramic groups

	Sample	Test statistic	Std. error	Std. test statistic	Sig.
**Thickness 3.0 mm LT**	**3.0 mm HT**	8.500	15.609	0.545	0.586
	**2.5 mm HT**	27.498	15.609	2.698	0.085
	**2.0 mm HT**	26.734	15.609	4.709	0.084
	**1.5 mm HT**	28.091	15.609	1.907	0.005
	**1.0 mm HT**	75.309	15.609	2.709	0.000
**Thickness 2.5 mm LT**	**3.0 mm HT**	28.479	15.609	2.179	0.113
	**2.5 mm HT**	2.667	15.609	0.171	0.864
	**2.0 mm HT**	12.409	15.609	4.309	0.073
	**1.5 mm HT**	2.209	15.609	2.609	0.023
	**1.0 mm HT**	48.409	15.609	3.409	0.000
**Thickness 2.0 mm LT**	**3.0 mm HT**	27.495	15.609	3.156	0.067
	**2.5 mm HT**	14.389	15.609	1.361	0.081
	**2.0 mm HT**	10.750	15.609	0.689	0.491
	**1.5 mm HT**	12.498	15.609	1.980	0.014
	**1.0 mm HT**	45.298	15.609	2.709	0.003
**Thickness 1.5 mm LT**	**3.0 mm HT**	30.798	15.609	2.798	0.034
	**2.5 mm HT**	17.491	15.609	3.456	0.012
	**2.0 mm HT**	9.587	15.609	3.298	0.042
	**1.5 mm HT**	19.708	15.609	1.263	0.207
	**1.0 mm HT**	47.260	15.609	2.471	0.000
**Thickness 1.0 mm LT**	**3.0 mm HT**	74.719	15.609	2.398	0.000
	**2.5 mm HT**	43.723	15.609	2.109	0.004
	**2.0 mm HT**	35.812	15.609	2.718	0.004
	**1.5 mm HT**	26.334	15.609	4.611	0.000
	**1.0 mm HT**	10.441	15.609	3.654	0.427

**Fig. 8:**
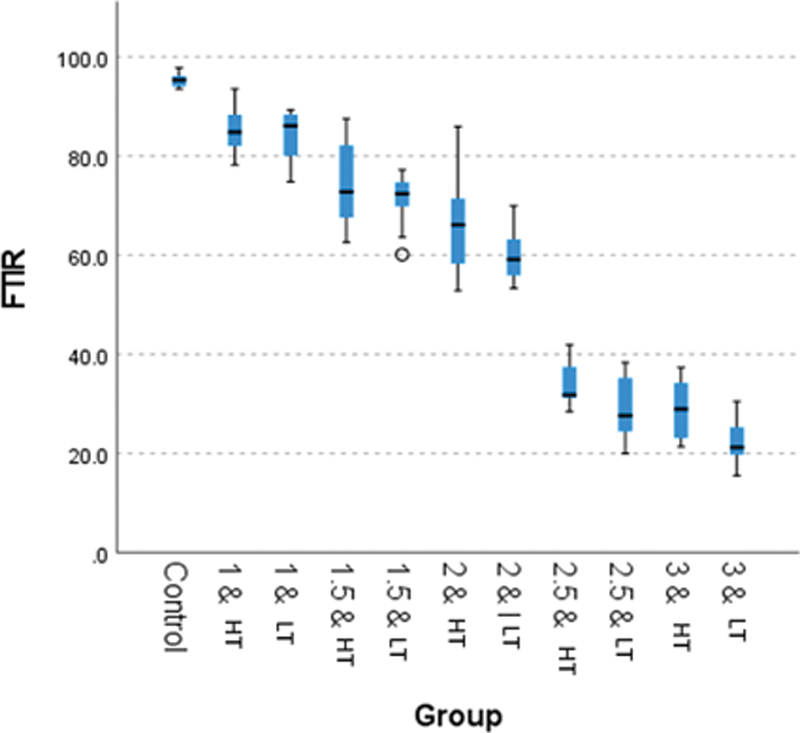
Boxplots of degree of conversion percentage (%DC) of 50-micron thickness light-cured resin cement polymerized without interposition of hybrid ceramic slab (control), and light-cured resin cement polymerized under 1.0, 1.5, 2.0, 2.5, and 3.0 mm of hybrid ceramic slabs of translucent and highly translucent types.

Comparison of the corresponding thickness of LT and HT Vita Enamic demonstrated that there was decreased DC% in LT as compared with HT Vita Enamic. Furthermore, analysis revealed no statistical significance between 1.0 mm HT and 1.0 mm LT hybrid ceramic ( *p* = 0.972), as the thickness of LT was increased to1.5, 2.0, 2.5, and 3.0 mm, the DC% was reduced in comparison to the corresponding HT thicknesses, but the differences did not reach statistical significance ( Table 14 and Fig. 8 ).

**Table 14:** Dunn's statistic results of comparison of percent degree of conversion (DC%) among high translucent (HT) and translucent (LT) Vita Enamic hybrid ceramic groups

	Sample	Test statistic	Std. error	Std. test statistic	Sig.
Thickness 3.0 mm LT	3.0 mm HT	13.833	15.615	0.886	0.376
	2.5 mm HT	12.467	15.615	1.109	0.264
	2.0 mm HT	50.679	15.615	4.065	0.002
	1.5 mm HT	65.721	15.615	4.310	0.003
	1.0 mm HT	94.958	15.615	6.081	0.000
Thickness 2.5 mm LT	3.0 mm HT	9.498	15.615	1.690	0.531
	2.5 mm HT	10.750	15.615	0.688	0.491
	2.0 mm HT	32.530	15.615	3.645	0.070
	1.5 mm HT	56.734	15.615	4.923	0.000
	1.0 mm HT	79.432	15.615	3.999	0.000
Thickness 2.0 mm LT	3.0 mm HT	43.209	15.615	3.154	0.004
	2.5 mm HT	30.439	15.615	4.078	0.078
	2.0 mm HT	10.091	15.615	2.012	0.076
	1.5 mm HT	22.299	15.615	2.943	0.009
	1.0 mm HT	32.600	15.615	1.980	0.000
Thickness 1.5 mm LT	3.0 mm HT	45.309	15.615	3.113	0.000
	2.5 mm HT	45.609	15.615	3.961	0.019
	2.0 mm HT	3.965	15.615	2.090	0.041
	1.5 mm HT	4.125	15.615	0.264	0.792
	1.0 mm HT	26.465	15.615	2.534	0.009
Thickness 1.0 mm LT	3.0 mm HT	79.054	15.615	2.908	0.000
	2.5 mm HT	69.187	15.615	4.509	0.000
	2.0 mm HT	13.465	15.615	1.980	0.009
	1.5 mm HT	20.517	15.615	1.987	0.018
	1.0 mm HT	0.542	15.615	0.035	0.972

## Discussion and Conclusion

The main purpose of this study was a comparative evaluation of the polymerization efficiency of light-curing resin cement containing the novel photoinitiator Ivocerin by the Vita Enamic hybrid ceramic, which has gained popularity as a veneering material for single-tooth treatment in recent years. Light-curing resin cements are usually preferred for their high esthetic properties and ultimate working time, with the possibility of not being fully cured under the restoration. 18 It has been reported that the recently introduced resin cements containing the novel dibenzoyl-germanium–based photoinitiator (Ivocerin) exhibit a higher DC% and higher color stability than resin cements containing camphorquinone (CQ). 22 Furthermore, a luting cement containing Ivocerin has a DC% of approximately 87%, whereas a cement containing CQ and tertiary amine has a DC% of approximately 44%. 29 It has also been reported that Ivocerin-based cement has a higher HV of approximately 47 HV, while CQ-based cement has 33 HV. 22 In this *in vitro* study, an attempt was made to simulate the clinical situations as far as possible. To simulate the clinical conditions for adhesive cement, where the cement is not exposed to air (except for the margins, unless an antioxidant gel is used), this study was designed to prevent polymerization inhibition by oxygen by covering the resin cement film with Mylar strips at the top and bottom similar to other studies. 30 In addition, the film thickness of the resin cement was controlled by using Mylar strips with a thickness of 50 µm as a spacer. The standardized cement thickness of 50 µm used in this study followed the recommendations of the International Organization for Standardization 4049:2019. 28 The performance of the light cure unit (LCU) in terms of irradiance and radiant exposure delivered to the sensor over a clinically relevant distance was evaluated. The mean incident irradiance of the light-curing unit used (Bluephase N LCU) was measured at 1,142 mW/cm ^2^ with a total energy delivered of 34.47 J/cm ^3^ .

According to Flury et al, 31 the light attenuation when light-curing the light-cured resin cement through 1.5, 3.0, and 6.0 mm thick ceramic restorations was more than 80, 95, and 99%, respectively. Theoretically, the irradiance of the light-curing device should be at least 500, 2,000, and 10,000 mW/cm ^2^ , respectively, when light-curing resin cement through 1.5, 3.0, and 6.0 mm thick ceramic restorations to achieve the minimum irradiance of 100 mW/cm ^2^ and a radiant exposure of 6 J/cm ^3^ recommended in the previous study of Li et al, 32 and the irradiation time must be more than 60 seconds. However, the irradiance of most commercially available light-curing devices is below 2,000 mW/cm ^2^ . 31 Therefore, light-cured resin cement should not be used for ceramic restorations with a thickness more than 3.0 mm in thickness.

It is still unclear in the literature whether light-cured resin cements can be properly cured by the novel hybrid ceramics. It is also unclear to what extent of the thickness or degree of translucency of these restorations may limit their use. Since the minimum thickness at the incisal edges and functional occlusal cusps must be 1.5 mm to ensure the longevity of the restoration, which is also recommended by the manufacturer of the Vita Enamic hybrid ceramic, and based on the fact that many *in vitro* studies have tested the depth of cure of polymerized resin cement at ceramic thicknesses up to 5.0 mm, 2 33 34 it was decided to investigate the effects of different thicknesses of Vita Enamic hybrid ceramic sections of the same shade 1M1 but with two different degrees of translucency on the polymerization efficiency of light-cured resin cement.

Microhardness testing indirectly provides useful information about the effectiveness of polymerization. 27 In addition, there is a good relationship between the DC% and microhardness, 35 although the relationship is complicated because microhardness is influenced not only by the DC% but also by the extent of crosslinking. 36 The results can also be affected by inadvertently placing the microhardness indenter directly on a filler particle. Another disadvantage of using microhardness is that the measurements cannot provide quantitative information about the proportion of double bonds that have undergone polymerization. On the other hand, FTIR is the most commonly used direct method to assess the DC%. It detects the vibrational frequency of carbon bonds and identifies functional groups at the molecular level in cured and uncured samples. 37 When the sample is placed on the ATR unit, it is exposed to the infrared spectrum. Molecules start to absorb infrared light and begin to vibrate. The detector measures the absorption and transmission of infrared light within the sample, at a specific wavelength, identifying functional groups and vibration peaks. 38 In resin-based materials, aliphatic C = C bonds were identified and recorded as 1,638 cm ^−1^ wavelength absorption peak values, while aromatic C = C double bonds were recorded as 1,609 cm ^−1^ wavelength absorption peak values. 39 This study employed both microhardness and FTIR tests to evaluate efficacy of polymerization of light-cured resin cement.

In our study, microhardness and FTIR tests were conducted 24 hours after curing, storing the samples in the dark and under dry conditions at 37°C, as proposed by Yan et al. They concluded that significant polymerization reactions were completed within 24 hours post-mix or post-light activation for all resin cements tested. 27

The results showed that increasing the thickness of the Vita Enamic slab affected the radiant exposure (J/cm ^3^ ) received by the light-cured resin cement (Variolink Esthetic). HT Vita Enamic slabs had delivered more energy to light-cured resin cement than their LT counterparts. However, statistical significance was demonstrated only in 1.5 mm thickness of hybrid ceramic, whereas for the rest of thicknesses the differences did not reach statistical significance. The first null hypothesis was rejected, as the thickness of Vita Enamic sections had a greater effect on radiant exposure than the translucency of the same shade of Vita Enamic hybrid ceramics. Babaier et al 39 found a strong inverse linear correlation between the translucency parameter of each CAD/CAM block material included in their study, and their thickness, consistent with the results of this study. In fact, this study found that a statistical significance was only present between the 1.5 mm HT slab and its LT counterpart.

Furthermore, the results showed that increased thickness of hybrid ceramic slabs reduced the degree of translucency.

Similarly, Egilmez et al 1 reported that the thickness of material significantly affected light irradiance, which in turn affected the energy reaching the resin material, consistent with the results of this study. They also showed that Vita Enamic exhibited the lowest values of transmitted light irradiance compared with other materials. 1 Lise et al 2 estimated the minimum required energy to obtain sufficient DC% and the maximum thickness of Vita Enamic through which resin cement can be cured. Furthermore, 2.1 J/cm ^3^ was considered the minimum radiant exposure, curing through a 1.9 mm thickness of Vita Enamic with a LCU power of 2,000 mW/cm ^2^ in high mode. In comparison, this study found radiant exposure to be 2.444 ± 0.124 J/cm ^3^ through a 2.5 mm thick HT slab, and 2.032 ± 0.1449 J/cm ^3^ through a 2.5 mm thick LT slab.

The shade was standardized to 1M1 (A1), for both HT and LT Vita Enamic hybrid ceramics, and the shade of the light resin cement was standardized to neutral, as light transmission characteristics are affected by the shade of materials and thickness of cement increments. 40 A light-cured resin cement film thickness of 50 µm was standardized for all samples.

Based on microhardness test results, the second null hypothesis was partially rejected, as this study showed that the thickness of Vita Enamic slabs affected the microhardness values of the light-cured resin cement Variolink Esthetic. However, there was no statistically significant difference in the microhardness values of light-cured resin cement among different degrees of translucency when corresponding thicknesses of Vita Enamic were compared.

Babaier et al 39 tested the hardness of Variolink Esthetic cement (LC and DC) 1 hour after light polymerization under various types of ceramic and composite CAD/CAM materials of different thicknesses. They reported that the hardness of resin luting discs was significantly affected by the type of CAD/CAM material and its thickness. Greater hardness was recorded for thicknesses of 1.0 and 2.0 mm, while the lowest hardness values were recorded for the 2.5 mm thick sections. 39 These results are consistent with this study, which found that thickness plays a significant role in microhardness values.

FTIR test results suggested that the thickness of Vita Enamic sections significantly affects the DC% of resin cement, while translucency has a minor but insignificant effect on DC%. The third null hypothesis was partially rejected, as interaction of different thicknesses of Vita Enamic slabs on the DC% of the light-cured resin cement Variolink Esthetic among HT and LT groups was prominent. The minimum clinically acceptable DC% has not been precisely established in the literature. In this study, DC% ranged from 95% for the control group to 22% for the 3.0 mm thick translucent (LT) 1M1 shade Vita Enamic. The DC% varies significantly among different ceramic materials. 41 42 Another study reported that cement polymerization depends on the shade of the restorative material, 43 where pigments in different shades can absorb light and affect polymerization. 43 However, in this study, the shade of the Vita Enamic hybrid ceramic used was standardized to 1M1 to preclude the effect of shade variation. Assessing changes in DC% during polymerization helps understand polymerization kinetics. Most post-irradiation polymerization occurs within the first few minutes or hours after light-curing, followed by a slower increase in DC% up to a maximum of 24 hours post-irradiation. 27 44 This suggests that all light-cured resin cement films showed an increase in DC% within 24 hours, regardless of the initial DC%. Additionally, resin cement films in this study were kept covered with Mylar strips to prevent the formation of an oxygen-inhibited layer as proposed by Gauthier et al. 30 The standardized cement thickness in our study is recommended by ISO 4049:2019, although many studies and a recent systematic review reported marginal adaptation of ceramic crowns to be less than or equal to 120 µm. 45 Increasing increments of resin cement may affect the depth of cure on its bottom surface. 16 46 Runnacles et al 46 evaluated the DC% of light-cured resin cement under different thicknesses of ceramics. They found that the DC% values of resin cements prepared under 0.5 and 1.0 mm thick ceramic samples were similar to the control group, but there was a significant reduction in DC% at thicknesses of 1.5 mm and above, 34 consistent with the results of this study. Despite the decreased DC% with increasing thickness of LT material compared with their HT counterparts, the differences did not reach statistical significance.

Barutcigil and Büyükkaplan 34 tested the DC% of RelyX light-cured and dual-cured resin cements, polymerized under varying thicknesses (0.5, 1.0, 1.5, and 2.0 mm) of Vita Enamic. They reported that despite the decreased DC% of the light-cured resin cement RelyX, as thickness increased, differences did not reach statistical significance. 34 Their results partially agree with the results of our current study.

In this study, increasing the thickness of Vita Enamic hybrid ceramic material decreased DC%. Despite the decreased DC% with increasing thickness of LT material compared with their HT counterparts, the differences did not reach statistical significance. Previous studies have reported that light-cured resin cements exhibit low DC%, especially for those prepared under ceramic restorations thicker than 1.0 mm. 47 48 In this study, light-cured resin cement showed a reduction in DC% with increasing thickness up to 2.0 mm, likely due to the increased thickness affecting light transmission and energy reaching the resin cement. Increasing thicknesses to 2.5 and 3 mm significantly reduced the DC% compared with the control group.

## Conclusion

Within limitations of this study, it can be concluded that:

- Increasing the thickness of Vita Enamic hybrid ceramic sections reduced light irradiance, received radiant exposure, HV, and the DC% of the light-cured resin cement Variolink Esthetic LC of neutral shade.
- Different translucency levels of Vita Enamic hybrid ceramic, namely translucent and highly translucent, had a small but nonsignificant effect on light irradiance, received radiant exposure, HV, and the DC% of the light-cured resin cement Variolink Esthetic LC of neutral shade.

## Clinical Significance

Clinically, it is recommended that dual-cured resin cement be used under restorations thicker than 2.0 mm.

## Clinical Relevance

The thickness of the Vita Enamic hybrid ceramic restoration is an important factor influencing the polymerization of the light-cured resin cement used to bond restorations and should be considered when bonding restorations.
